# Battle of the giants: KDIGO vs. Cochrane — who is the winner in the fight for blood pressure targets in chronic kidney disease?

**DOI:** 10.1590/2175-8239-JBN-2025-0335en

**Published:** 2026-04-03

**Authors:** Cibele Isaac Saad Rodrigues, Ana Flávia Moura, Rodrigo Bezerra

**Affiliations:** 1 Pontifícia Universidade Católica de São Paulo, Faculdade de Ciências Médicas e da Saúde, Sorocaba, SP, Brazil.; 2 Escola Bahiana de Medicina e Saúde Pública, Salvador, BA, Brazil.; 3 Universidade de Pernambuco, Pronto Socorro Cardiológico de Pernambuco, Recife, PE, Brazil.

**Keywords:** Hypertension, Renal Insufficiency, Chronic, Arterial Pressure, Systematic Review

## Abstract

Hypertension (HTN) in chronic kidney disease (CKD) is often regarded as the main villain. It represents the leading cause of dialysis-dependent CKD in Brazil and the second most common worldwide, yet it is also a frequent consequence of renal disorders that induce secondary HTN. In other words, HTN in CKD is a double-edged sword—causing disease progression when primary and aggravating existing injury when secondary, through multiple pathophysiological mechanisms. The most frequently cited blood pressure targets for CKD management in the current scientific literature come from two major authorities in evidence-based recommendations: the 2024 KDIGO (Kidney Disease: Improving Global Outcomes) clinical practice guidelines and the 2024 Cochrane Library systematic review and meta-analysis. This article aims to examine and compare the blood pressure targets proposed for CKD by these two leading institutions.

## Graphical abstract

**Figure:**
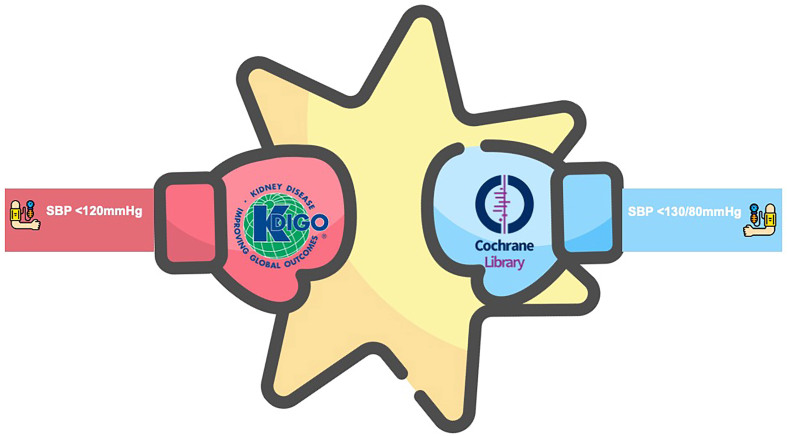
Abbreviation – SBP: Systolic blood pressure.

## Introduction

Hypertension (HTN) is the leading cause of advanced chronic kidney disease (CKD) in Brazil^1^ and the second most common worldwide^2^. Conversely, it may also occur as a secondary manifestation of diverse renal pathologies. In many cases, it presents as resistant or refractory HTN, requiring combination therapy with multiple synergistic antihypertensive agents at maximum tolerated doses and, preferably, with pleiotropic properties^3,4^. In other words, HTN in CKD represents a bidirectional condition: it is both a causal factor and a determinant of CKD progression and, when secondary to renal dysfunction, it worsens pre-existing lesions through multiple pathophysiological mechanisms, including sodium retention, hyperactivation of the renin–angiotensin–aldosterone system (RAAS), and endothelial dysfunction^5,6^. Moreover, HTN is the leading modifiable cause of cardiovascular disease and death among patients with CKD^7,8^.

Despite the central role of HTN in both the development and progression of CKD, patients with advanced stages of the disease are frequently excluded from randomized controlled trials (RCTs) evaluating HTN management and target blood pressure (BP) levels. Consequently, clinical decisions for these patients have relied on evidence derived from small studies, highly selected populations, and substantial extrapolation of outcomes observed in the general population^9^. Despite recent updates in clinical practice guidelines for HTN management in CKD, there is still no consensus regarding the optimal BP targets for this population, a gap that is largely attributed to the paucity of robust RCTs capable of supporting consistent and high-certainty recommendations.

## Discussion

The most frequently cited BP targets for CKD management in the current scientific literature come from two major authorities in the field of nephrology: KDIGO (Kidney Disease: Improving Global Outcomes)^10^ and the Cochrane Library^11^.

**Figure 1:**
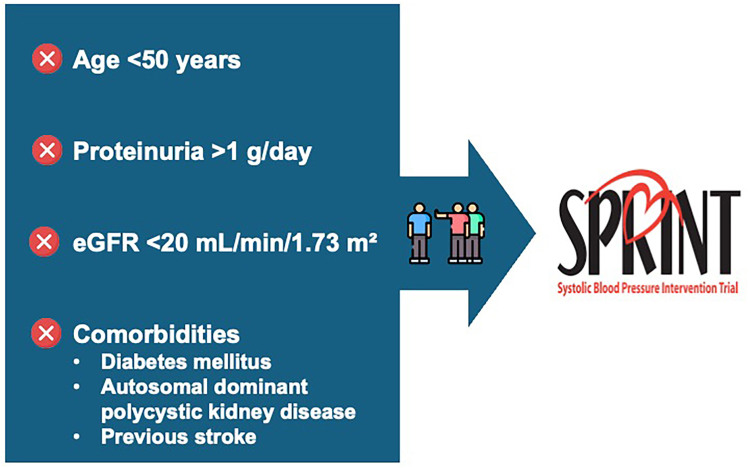
Exclusion criteria used in the SPRINT trial^12^.

**Figure 2:**
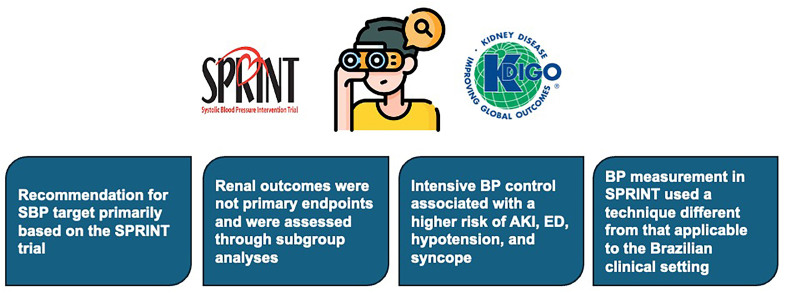
Considerations regarding the SPRINT trial that may influence the blood pressure targets recommended by KDIGO.

It is well established that the KDIGO recommendations^10^ are firmly aligned with the SPRINT trial^12^ and its secondary analysis of renal outcomes. However, several important considerations should be noted. Briefly, the SPRINT trial included numerous exclusion criteria (Figure 1) that are common among patients with CKD and highly relevant to the evaluation of this population (Figure 2).

Abbreviations – eGFR: estimated glomerular filtration rate.

Abbreviations – BP: blood pressure; SBP: systolic blood pressure; AKI: acute kidney injury; ED: electrolyte disturbance.

A careful examination of the KDIGO recommendation^10^ – which states: *“We suggest that adults with HTN and CKD be treated to a target SBP <120 mmHg, when tolerated, using automated office BP measurement (2B)”* – reveals at least six key points that warrant reflection.

1. **Limited evidence and reliance on a single trial:** The KDIGO^10^ recommendation of a systolic BP (SBP) target of <120 mmHg is grounded primarily in the CKD subgroup analysis of the SPRINT trial^12^, which excluded patients with diabetic kidney disease, the most common cause of CKD worldwide and the second most common in Brazil. In addition, SPRINT excluded patients with autosomal dominant polycystic kidney disease, proteinuria >1.0 g/day, a history of prior stroke, or an estimated glomerular filtration rate (eGFR) <20 mL/min/1.73 m^2^. These exclusions substantially limit the generalizability of the findings to the broader CKD population, particularly for patients with diabetes, glomerulopathies characterized by albuminuria, or more advanced stages of CKD^13,14,15^.
2. **Technique used for BP measurement:** In the SPRINT trial^12^, BP was measured using an automated and often unattended protocol. An automatic device recorded three consecutive readings at one-minute intervals after five minutes of rest, with the observer remaining outside the room during measurement in most centers. The impact of this methodology was confirmed in a SPRINT substudy^16^ in which ambulatory BP monitoring (ABPM) demonstrated that mean daytime values were 7 mmHg higher than office readings in the intensive-treatment group and 3 mmHg higher in the standard-treatment group. This finding is unusual, as ABPM values are typically lower than office measurements. These results suggest that unattended automated office BP measurements tend to underestimate BP compared with the standard attended technique, which has important implications for the interpretation and clinical application of the BP targets analyzed in SPRINT. Consequently, applying a target SBP <120 mmHg to non-standardized measurements may lead to misclassification, overtreatment, and additional risks, as no validated correction factor exists to translate values obtained under non-standardized conditions^13,15,16^.
3. **Risk of adverse events in vulnerable populations:** Patients with advanced CKD (stages 4 and 5; eGFR <30 mL/min/1.73 m^2^), older adults (>60 years), the very elderly (>80 years), those with frailty, or those with multiple comorbidities may be at increased risk of serious adverse events when more intensive BP targets are pursued. These include hypotension and/or orthostatic hypotension, falls secondary to presyncope or syncope, fractures, renal hypoperfusion with acute kidney injury (AKI), electrolyte disturbances, and complications related to polypharmacy. The KDIGO guideline does not sufficiently individualize BP targets for these subgroups, which may be potentially harmful, although it does include a practical remark: *“Consider less intensive BP-lowering therapy in people who are frail, have a high risk of falls and fractures, limited life expectancy, or symptomatic postural hypotension.”* ^13,15^
4. **Inconsistency with other international guidelines:** The BP targets suggested by KDIGO^10^ (SBP <120 mmHg) are lower than those recommended by all other major guidelines – Brazilian, American, and European –, creating confusion among clinicians managing patients with HTN and CKD and hindering the harmonization of care^14,15^. Table 1 summarizes the BP targets proposed by the different guidelines^8,11,17,18,19,20,21,22,23,25^.
5. **Lack of robust evidence for renal benefit:** Although there is consistent evidence that lower BP targets reduce cardiovascular events across nearly all major HTN outcomes, studies evaluating potential benefits for CKD progression are far less conclusive. Significant reductions in CKD progression have been demonstrated only among patients with substantial proteinuria^8,18^.
6. **Challenges related to adherence and polypharmacy:** Achieving lower BP targets often requires the use of multiple antihypertensive agents, increasing the risk of drug–drug interactions and adverse effects such as poor adherence and therapeutic complexity. These issues are particularly relevant among patients with markedly reduced kidney function and among older or very elderly individuals, populations that are growing substantially worldwide, including those receiving renal replacement therapy^15,26^.

**Table 1:** Major guidelines on blood pressure targets for non-dialysis chronic kidney disease

Guidelines	Year	Blood Pressure Target (mmHg)CKD with or without Proteinuria
Joint National Committee	JNC8-2014^17^	<140/<90
American College of Cardiology and American Heart Association	ACC/AHA-2018^18^ ACC/AHA-2025^8^	<130/<80 PAS < 130
European Society of Cardiology and European Society of Hypertension	ESC/ESH-2018^19^	PAS < 130–140
International Society of Hypertension	ISH-2020^20^	<130/<80 <140/<90 (elderly)
World Health Organization	WHO-2021^21^	PAS < 130
European Society of Hypertension	ESH-2023^22^	PAS < 130–140
Kidney Disease: Improving Global Outcomes	KDIGO-2021^23^/2024^10^	PAS < 120
European Society of Cardiology	ESC-2024^24^	PAS 120–129 and PAD 70–79
Brazilian Hypertension Guidelines	BHG-2025^25^	<130/80

Abbreviations – BP, blood pressure (mmHg); SBP, systolic blood pressure; DBP, diastolic blood pressure; CKD, chronic kidney disease; JNC8, Eighth Joint National Committee; ACC/AHA, American College of Cardiology/American Heart Association; ESC/ESH, European Society of Cardiology/European Society of Hypertension; ISH, International Society of Hypertension; WHO, World Health Organization; KDIGO, Kidney Disease: Improving Global Outcomes; BHG, Brazilian Hypertension Guidelines.

### Systematic Review and Meta-Analysis Published in the Cochrane Library

The systematic review by Erviti et al.^11^ recommends a different BP target for patients with HTN and CKD. Data from six RCTs included in this review, encompassing 7,348 participants, including those from the SPRINT trial^12^, showed that more intensive BP targets (≤130/80 mmHg or SBP <120 mmHg) did not confer clear benefits for all-cause mortality (RR 0.90; 95% CI, 0.76–1.06), cardiovascular mortality, cardiovascular events (RR 1.00; 95% CI, 0.87–1.15), or progression to advanced kidney disease (RR 0.94; 95% CI, 0.80–1.11) when compared with standard targets (140–160/90–100 mmHg). Moreover, as previously noted, intensive BP control may increase the risk of adverse events and the need for multiple medications. No relevant differences were observed in renal outcomes such as doubling of serum creatinine levels or ≥50% reduction in eGFR^11,27^.

Three studies were publicly funded, two were pri­vately funded, and one had mixed public–private support. All studies provided low to moderate certainty of evidence for the outcomes of interest, with no significant differences in serious adverse events or treatment discontinuations due to side effects.

However, the Cochrane review^11^ emphasizes that, in patients with CKD, the benefits of stricter BP targets remain uncertain and may not justify the additional medication burden and the risk of adverse effects. The review underscores the need for caution and for individualized BP targets, considering patient tolerance, comorbidities, and the risk of adverse events.

## Conclusion

A cardiovascular- and kidney risk–based approach to HTN management in CKD may be as valuable as the target-based strategies described in the various guidelines (Table 1). RCTs are still needed to determine the optimal BP goal at which benefits outweigh risks for most individuals, ideally supported by reliable and standardized parameters^28^.

New studies are strongly warranted to fill this knowledge gap. They should include eligibility criteria that better reflect real-world clinical practice, such as patients with advanced CKD (stages 4–5), diverse age groups, sexes, socioeconomic and cultural backgrounds, multiple etiologies of CKD, and coexisting comorbidities.

Although intensive BP lowering (SBP <120 mmHg) may reduce cardiovascular events in specific subgroups, such as those at higher cardiovascular risk, the renal benefits remain limited and inconsistent^29,30^, and the risk of adverse events may outweigh potential gains in vulnerable populations.

It is also important to emphasize that standardized BP measurement is rarely performed in Brazil and in many other countries; thus, comparisons with standardized trial measurements may lead to dangerous misinterpretations with potentially unpredictable consequences.

In conclusion, in this “battle of giants” between KDIGO^10^ and the Cochrane^11^, we favor adherence to the 2025 Brazilian Hypertension Guidelines^24^, considering the Brazilian context and the limitations discussed above. Ultimately, in this ongoing debate over BP targets in CKD, the true winner should be the patient – each and every one of them.
