# Supplementary material for: Battle of the giants: KDIGO vs. Cochrane — who is the winner in the fight for blood pressure targets in chronic kidney disease?
Source: J Bras Nefrol. 2026 Apr 3;48(2):e20250335. doi: 10.1590/2175-8239-JBN-2025-0335en (PMC13061537; doi:10.1590/2175-8239-JBN-2025-0335en)
Supplement: Translation

# Translation: Briga de gigantes: KDIGO vs. Cochrane — quem é o vitorioso na luta pela meta de pressão arterial na doença renal crônica?

**DOI:** 10.1590/2175-8239-JBN-2025-0335pt

**Authors:** Cibele Isaac Saad Rodrigues, Ana Flávia Moura, Rodrigo Bezerra

**Affiliations:** 1 Pontifícia Universidade Católica de São Paulo, Faculdade de Ciências Médicas e da Saúde, Sorocaba, SP, Brasil.; 2 Escola Bahiana de Medicina e Saúde Pública, Salvador, BA, Brasil.; 3 Universidade de Pernambuco, Pronto Socorro Cardiológico de Pernambuco, Recife, PE, Brasil.

**Keywords:** Hipertensão, Insuficiência Renal Crônica, Pressão Arterial, Revisão Sistemática

## Abstract

A hipertensão arterial (HA) na doença renal crônica (DRC) é considerada um vilão, sendo a principal causa de DRC dialítica no Brasil e a segunda maior no mundo. Por outro lado, é também vítima da imensa maioria das doenças que afetam os rins e determinam HA secundariamente. Ou seja, a HA na DRC é uma faca de dois gumes, pois é um fator causal e determinante de progressão e, quando consequência da doença renal, agride e piora lesões pré-existentes devido a múltiplos mecanismos fisiopatogênicos. As recomendações para as metas de tratamento na DRC mais citadas na literatura científica atual incluem dois gigantes na elaboração de recomendações baseadas em evidências: as diretrizes do KDIGO – *Kidney Disease: Improving Global Outcomes* – e a revisão sistemática com meta-análise da Cochrane Library, ambas publicadas em 2024. Este artigo visa analisar as metas de pressão arterial escolhidas na DRC por essas instituições.

## Introdução

A hipertensão arterial (HA) é a principal causa de doença renal crônica (DRC) avançada no Brasil^1^ e a segunda maior no mundo^2^. Por outro lado, também pode manifestar-se como consequência de diversas afecções renais, configurando-se como HA secundária. Em muitos casos, costuma apresentar-se como resistente ou refratária, exigindo o uso combinado de múltiplos medicamentos anti-hipertensivos sinérgicos, em doses máximas toleradas e, preferencialmente, com propriedades pleiotrópicas^3,4^. Ou seja, a HA na DRC consiste em uma condição de dupla via: é fator causal e determinante da progressão da DRC e, quando é consequência da disfunção renal, agrava as lesões pré-existentes devido a múltiplos mecanismos fisiopatogênicos, como retenção de sódio, hiperatividade do sistema renina-angiotensina-aldosterona (SRAA) e disfunção endotelial^5,6^. Além disso, a HA é a principal causa modificável de doenças cardiovasculares e de óbito em pacientes com DRC^7,8^.

Pessoas com DRC em estágios mais avançados são frequentemente enquadradas nos critérios de exclusão para ensaios clínicos randomizados (ECRs) que avaliam o manejo da HA e as metas de PA a serem alcançadas. As decisões clínicas para esses pacientes baseiam-se em evidências provenientes de estudos com amostras reduzidas, populações específicas e significativa extrapolação dos desfechos encontrados na população em geral^9^. Apesar das atualizações recentes nas diretrizes sobre o manejo da hipertensão arterial HA na DRC, ainda não há consenso quanto às metas ideais para essa população — uma lacuna atribuída à escassez de ECRs robustos que permitam recomendações consistentes e de alta confiabilidade.

## Discussão

As recomendações para as metas de tratamento na DRC mais citadas na literatura científica atual incluem dois gigantes na elaboração de recomendações na área de nefrologia: o KDIGO — *Kidney Disease: Improving Global Outcomes* ^10^ e a *Cochrane Library* ^11^.

É certo que as recomendações do KDIGO^10^ estão firmemente alinhadas com o estudo SPRINT^12^ e sua análise secundária de desfechos renais. No entanto, cabem algumas ponderações descritas a seguir que, resumidamente, incluem muitos critérios de exclusão (Figura 1), comuns na população com DRC e relevantes para a avaliação desses pacientes (Figura 2).

**Figura 1:** Critérios de exclusão utilizados no estudo SPRINT^12^.

Abreviaturas – TFGe: taxa de filtração glomerular estimada; AVC: acidente vascular cerebral.

**Figura 2:** Considerações sobre o estudo SPRINT^12^ que podem impactar as metas preconizadas pelo KDIGO.

Abreviaturas – PA: pressão arterial; PAS: pressão arterial sistólica; IRA: injúria renal aguda; DHE: distúrbio hidroeletrolítico.

Numa análise cuidadosa da recomendação proposta pelo KDIGO^10^ — que diz: *“Sugere-se que adultos com HA e DRC sejam tratados com a meta de PAS <120 mmHg, quando tolerado, utilizando a medida automatizada de PA de consultório (2B)”* — temos pelo menos seis pontos de reflexão a considerar.

1. **Evidências limitadas e dependência de um único estudo**: A recomendação da meta de PA sistólica (PAS) <120 mmHg pelo KDIGO^10^ é fundamentada principalmente na análise do subgrupo de DRC do estudo SPRINT^12^, que excluiu pacientes com doença renal diabética, a causa mais comum de DRC no mundo e a segunda no Brasil. Além disso, excluiu pacientes com doença renal policística do adulto, proteinúria >1,0 g/dia, com história de acidente vascular cerebral (AVC) prévio ou taxa de filtração glomerular estimada (TFGe) <20 mL/min/1,73 m^2^. Essa escolha limita a generalização dos resultados para a população global de DRC, especialmente para pacientes com diabetes, glomerulonefrites que cursam com albuminúria, ou em estágios mais avançados da doença^13,14,15^.
2. **Técnica utilizada na medida da PA**: No estudo SPRINT^12^, a PA foi medida de forma automatizada e frequentemente desacompanhada, utilizando-se um dispositivo automático que registrava três leituras consecutivas, com intervalo de um minuto, após cinco minutos de repouso, sendo que o examinador não permanecia na sala durante a medição em grande parte dos centros. O impacto dessa metodologia foi confirmado em um subestudo do SPRINT^16^, no qual a monitorização ambulatorial da PA (MAPA) demonstrou que os valores diurnos da MAPA foram, em média, 7 mmHg mais elevados que as leituras de consultório no grupo intensivo e 3 mmHg mais elevados no grupo padrão, achado incomum, já que habitualmente as pressões obtidas pela MAPA tendem a ser inferiores às medidas de consultório. Esses resultados sugerem que a medida automatizada desacompanhada tende a subestimar a PA quando comparada à técnica padrão de medida no consultório, tendo implicações importantes para a interpretação e a aplicação clínica das metas de pressão arterial analisadas no estudo SPRINT. Assim, aplicar o alvo de PAS <120 mmHg a medidas não padronizadas pode resultar em diagnóstico impreciso, tratamento excessivo e riscos adicionais, pois não existe um fator de correção validado para converter valores obtidos em ambientes não padronizados^13,15,16^.
3. **Risco de eventos adversos em populações vulneráveis**: Pacientes com DRC avançada, nos estágios 4 e 5 (TFG <30 mL/min/1,73 m^2^), idosos (>60 anos) ou muito idosos (>80 anos), frágeis ou com múltiplas comorbidades, podem apresentar maior risco de eventos adversos graves ao se tentar atingir metas mais rigorosas de PA, tais como hipotensão e/ou hipotensão postural, quedas secundárias à lipotimia ou mesmo síncope, fraturas, hipofluxo renal com injúria renal aguda (IRA), distúrbios eletrolíticos e polifarmácia. A diretriz do KDIGO^10^ não individualiza suficientemente as metas para esses subgrupos, o que pode ser potencialmente prejudicial, embora aponte um ponto prático: *“Considere uma terapia anti-hipertensiva menos intensiva em pessoas com fragilidade, alto risco de quedas e fraturas, com baixa expectativa de vida ou hipotensão postural sintomática”* ^13,15^.
4. **Inconsistência com outras diretrizes internacionais**: As metas sugeridas pelo KDIGO^10^ (PAS <120 mmHg) são mais baixas do que as recomendadas por todas as outras diretrizes relevantes — brasileiras, americanas e europeias — gerando confusão entre profissionais que assistem pessoas com HA e DRC e dificultando a harmonização do cuidado^14,15^. A Tabela 1 traz um resumo das metas preconizadas pelas diferentes diretrizes^8,11,17,18,19,20,21,22,23,25^.
5. **Ausência de evidências robustas para benefício renal**: Embora existam evidências de redução de eventos cardiovasculares com metas mais baixas de PA para praticamente todos os desfechos duros em HA, os estudos que analisaram possíveis benefícios na progressão da DRC são bem menos consistentes e demonstram redução significativa da progressão da DRC apenas em pacientes com proteinúria significativa^8,18^.
6. **Desafios na adesão e polifarmácia**: Atingir metas mais baixas de PA frequentemente exige a associação de múltiplos medicamentos, aumentando o risco de interações medi­camentosas e efeitos adversos, como baixa adesão ao tratamento e maior complexidade terapêutica, especialmente em pacientes com função renal muito reduzida e em idosos ou muito idosos, populações que crescem consideravelmente em todo o mundo, incluindo aqueles em terapia renal substitutiva^15,26^.

**Tabela 1:** Principais diretrizes que indicam metas de pressão arterial para pacientes com doença renal crônica em tratamento conservador

Diretrizes	Ano	Meta de Pressão Arterial (mmHg)DRC com ou sem Proteinúria
Joint National Committee	JNC8-2014^17^	<140/<90
American College of Cardiology e American Heart Association	ACC/AHA-2018^18^ ACC/AHA-2025^8^	<130/<80 PAS < 130
European Society of Cardiology e European Society of Hypertension	ESC/ESH-2018^19^	PAS < 130–140
International Society of Hypertension	ISH-2020^20^	<130/<80 <140/<90 (idosos)
Organização Mundial da Saúde	OMS-2021^21^	PAS < 130
European Society of Hypertension	ESH-2023^22^	PAS < 130–140
Kidney Disease: Improving Global Outcomes	KDIGO-2021^23^/2024^10^	PAS < 120
European Society of Cardiology	ESC-2024^24^	PAS 120–129 e PAD 70–79
Diretriz Brasileira de Hipertensão 2025	DBHA-2025^25^	<130/80

Abreviaturas – PA: pressão arterial (mmHg); PAS: pressão arterial sistólica; DRC: doença renal crônica; JNC8: Eighth Joint National Committee; ACC/AHA: American College of Cardiology/American Heart Association; ESC/ESH: European Society of Cardiology/European Society of Hypertension; ISH: International Society of Hypertension; OMS: Organização Mundial da Saúde; KDIGO: Kidney Disease: Improving Global Outcomes; DBHA: Diretriz Brasileira de Hipertensão Arterial.

### Revisão Sistemática com Meta-Análise da Cochrane Library

A revisão sistemática de Erviti et al.^11^ recomenda uma meta diferente de PA para pacientes com HA e DRC. Dados dos seis ECRs analisados nesta revisão, incluindo o SPRINT^12^, somando 7.348 participantes, mostraram que metas mais rigorosas de PA (≤130/80 mmHg ou PAS <120 mmHg) não trazem benefícios inequívocos em mortalidade total (RR 0,90; IC 95% 0,76–1,06) ou cardiovascular, eventos cardiovasculares (RR 1,00; IC 95% 0,87–1,15) ou na progressão para doença renal avançada (RR 0,94; IC 95% 0,80–1,11) quando comparadas às metas padrão (140–160/90–100 mmHg). Além disso, conforme já citado, um controle mais intensivo da PA pode aumentar o risco de eventos adversos e a necessidade de mais medicamentos. Ademais, não houve diferença relevante em desfechos renais, como duplicação da creatinina ou redução ≥50% da TFG^11,27^.

Três estudos foram financiados com recursos públicos, dois com recursos privados e um com financiamento público e privado. Todos os estudos apresentaram baixa a moderada certeza da evidência para os desfechos de interesse, e não houve diferença significativa em eventos adversos graves ou na descontinuação do estudo por efeitos colaterais.

No entanto, a revisão Cochrane^11^ destaca que, para pacientes com DRC, os benefícios dessas metas mais baixas são incertos e podem não justificar o aumento da carga medicamentosa e do risco de efeitos adversos.

A revisão sistemática sugere precaução e individualização da meta de PA, considerando a tolerância comorbidades e o risco de eventosadversos.

## Conclusão

Uma abordagem da HA na DRC, baseada em risco cardiovascular e renal, pode ser tão valiosa quanto as metas descritas em diferentes diretrizes (Tabela 1). Ensaios clínicos randomizados ainda serão necessários para identificar a melhor meta de PA em que os benefícios superem os riscos para a maioria dos indivíduos e que, igualmente, seja pautada em parâmetros confiáveis^28^.

Novos estudos são muito bem-vindos para preencher essa lacuna de conhecimento e devem ter critérios de inclusão mais alinhados com a realidade da prática clínica – pessoas com DRC avançada (estágios 4 e 5), diferentes faixas etárias, sexos e condições socioeconômicas e culturais, diversas etiologias de DRC e presença de múltiplas comorbidades.

Embora o tratamento intensivo da PA (PAS <120 mmHg) possa reduzir eventos cardiovasculares em subgrupos específicos, como pacientes com maior risco cardiovascular, os benefícios renais são limitados e contraditórios^29,30^, e o risco de eventos adversos pode superar os ganhos em populações vulneráveis.

Vale ressaltar que a medida da PA de modo padronizado é pouco realizada no Brasil e em muitos países do mundo, e comparações com medidas padronizadas podem nos levar a erros perigosos, com consequências nem sempre previsíveis.

Finalizando, nesta luta de gigantes, como o KDIGO^10^ e a Cochrane^11^, preferimos seguir as Diretrizes Brasileiras de Hipertensão 2025^24^, considerando o contexto brasileiro e as limitações apresentadas.

É certo que, nesta “luta” pelas metas de PA na DRC, o grande vencedor deve ser o paciente: todos e cada um deles!
